# A Bibliometric Analysis of 14,822 Researches on Myocardial Reperfusion Injury by Machine Learning

**DOI:** 10.3390/ijerph18158231

**Published:** 2021-08-03

**Authors:** Chan Li, Zhaoya Liu, Ruizheng Shi

**Affiliations:** 1 Department of Cardiovascular Medicine, Xiangya Hospital, Central South University, Changsha 410008, China; chanli@csu.edu.cn; 2 Department of the Geriatrics, The Third Xiangya Hospital, Central South University, Changsha 410013, China; liuzhaoya@csu.edu.cn

**Keywords:** myocardial reperfusion injury, bibliometric analysis, LDA analysis, machine learning, MeSH term

## Abstract

Myocardial ischemia is the major cause of death worldwide, and reperfusion is the standard intervention for myocardial ischemia. However, reperfusion may cause additional damage, known as myocardial reperfusion injury, for which there is still no effective therapy. This study aims to analyze the landscape of researches concerning myocardial reperfusion injury over the past three decades by machine learning. PubMed was searched for publications from 1990 to 2020 indexed under the Medical Subject Headings (MeSH) term “myocardial reperfusion injury” on 13 April 2021. MeSH analysis and Latent Dirichlet allocation (LDA) analyses were applied to reveal research hotspots. In total, 14,822 publications were collected and analyzed in this study. MeSH analyses revealed that time factors and apoptosis were the leading terms of the pathogenesis and treatment of myocardial reperfusion injury, respectively. In LDA analyses, research topics were classified into three clusters. Complex correlations were observed between topics of different clusters, and the prognosis is the most concerned field of the researchers. In conclusion, the number of publications on myocardial reperfusion injury increases during the past three decades, which mainly focused on prognosis, mechanism, and treatment. Prognosis is the most concerned field, whereas studies on mechanism and treatment are relatively lacking.

## 1. Introduction

Myocardial ischemia is the major cause of death and disability, accounting for 16% of total death worldwide [1]. Myocardial ischemia is caused by thrombotic complications followed by coronary atherosclerotic plaque, which leads to biochemical and metabolic changes and eventually cardiomyocyte death. In patients with acute myocardial infarction (AMI) whose coronary artery is completely occluded, this kind of cell death is further exacerbated and endangers their lives. To date, the application of thrombolytic therapy or percutaneous coronary intervention (PCI) is the most effective therapy for myocardial ischemia especially acute ST-segment elevation myocardial infarction (STEMI) [2]. However, reperfusion may cause additional myocardial injury such as myocardial stunning, arrhythmia, microvascular obstruction, and cardiomyocyte death, known as myocardial reperfusion injury. The occurrence of myocardial reperfusion injury greatly reduces the beneficial effects of reperfusion therapy, increases the infarct size and worsens the prognosis [3]. Despite an enormous number of studies having focused on the mechanism of myocardial reperfusion injury, such as oxidative stress, inflammation, and calcium overload, there is still no effective therapy for myocardial reperfusion injury [4]. Recently, a number of novel therapeutic strategies such as ischemic pre-, per-, and post-conditioning [5] and therapeutic hyper-oxemia [6] have been proposed and shown to be effective in experimental studies. However, their efficacy in the clinic is limited, which may be due to an incomplete understanding of their underlying mechanisms [5,6]. Therefore, the prevention and treatment of myocardial reperfusion injury, as well as their underlying mechanisms, are still challenges for physicians and demand to be investigated urgently.

In order to better understand research hot topics and provide research directions in the future, it is of great importance to systematically analyze the priorities and trends of previous researches. Most of the researches about myocardial reperfusion injury were reported in the form of publications, which increased gradually with the development of the publishing industry. Importantly, the number of publications in a field is closely related to the degree of public attention and reflects research interest. Bibliometric analysis is a method to quantitatively analyze academic literatures and has been widely used to reveal research trends in cardiovascular diseases, including myocardial infarction [7], atrial fibrillation [8], and hypertension [9]. Nevertheless, bibliometric analysis on myocardial reperfusion injury is still urgent. In addition, previous bibliometric analysis mainly focused on high-cited publications due to lack of effective methods dealing with large amounts of literatures [10,11,12]. Natural language processing (NLP), a series of machine learning methods for analyzing human language, has been employed to process medical information in recent years [13]. Latent Dirichlet allocation (LDA) is a classic method of NLP and has been applied in bibliometric analysis to extract specific topics from a large number of publications by machine learning [14,15]. It creates a characteristic term glossary based on the frequency of the coexisted vocabulary and classifies the articles into different topics based on the frequency of these characteristic terms appearing in each publication. This method makes it possible to carry out a comprehensive bibliometric analysis on myocardial reperfusion injury.

In this study, machine learning method LDA and bibliometric analysis were conducted to reveal the research topics of scientific publications related to myocardial reperfusion injury during the past three decades. Our results may contribute to better understanding recent research hot topics and provide potential directions for future studies.

## 2. Materials and Methods

### 2.1. Data Collection

PubMed was searched for publications on 13 April 2021, using the Medical Subject Headings (MeSH) term “myocardial reperfusion injury”. Publications during 1990 and 2020 were downloaded in the format of PubMed. Information such as publication year, publication type, country, affiliation, MeSH term, and abstract were extracted by R package “Bibliometrix” [16].

### 2.2. MeSH Analyses

MeSH terms with occurrence more than 25 times were included in MeSH analyses according to previous studies [14]. The cumulative occurrence of each MeSH term obtained by R package (R Core Team, Vienna, Austria) was visualized by Excel (Redmond, WA, USA).

### 2.3. LDA Analyses

LDA is a topic modeling method that can identify the characteristics of a large number of texts. LDA analyses were conducted to obtain research topics with greater specificity from the abstract of the articles using Python [14,17]. LDA analyses assume that an article is made up of several topics, which can be identified by the following steps. Firstly, a characteristic term glossary was created based on how often the vocabulary words coexisted in the abstract of the downloaded articles. Then, if several characteristic terms frequently appear together in multiple publications, such as “imaging”, “iron”, “resonance”, “mri” and “magnetic”, LDA would recognize these terms as a topic. Next, since these topics were a set of words, we need to name them manually. For instance, when we see a topic including words such as “imaging”, “iron”, “resonance”, “mri”, and “magnetic”, we can name this topic “magnetic resonance imaging”. MeSH terms were used as a reference for naming these topics. The criteria of topic number selection were based on previous studies, and 50 research topics were classified in this study [14]. For each article, the top two research topics were identified based on the frequency of the relevant characteristic terms. Finally, the Louvain algorithm was used to build a network and illustrate the relationship between these topics.

R codes and Python codes used in this article were obtained from GitHub [18] (https://github.com/yan-wen0614/Medicine-Bibliometric-Analysis, accessed on 20 May 2021) and provided as File 1 in Supplementary Materials. Gephi (https://gephi.org/, accessed on 20 May 2021) was applied to conduct the topic network [19].

## 3. Results

### 3.1. Characteristics of the Publications

**Figure 1:**
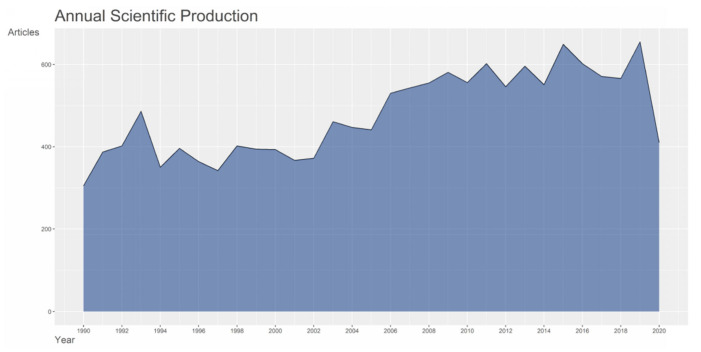
Number of articles published per year.

**Figure 2:**
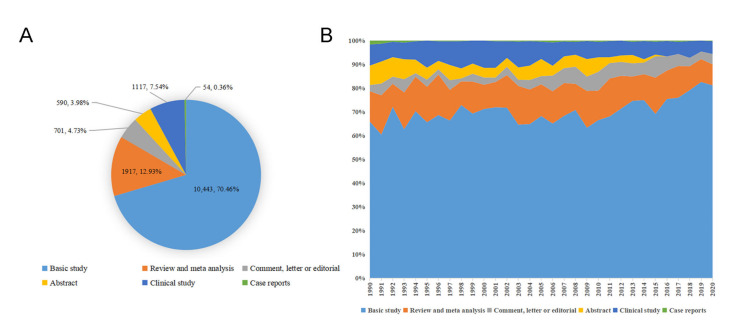
Distribution of article type. (**A**) total number and proportion of different article types in the past three decades; (**B**) distribution of different article types per year.

By searching MeSH term “myocardial reperfusion injury”, 14,822 publications were downloaded from PubMed and included in this study. The number of publications fluctuated around 420 per year from 1990 to 2005 and increased abruptly in 2006. The average number of publications was 579 per year from 2006 to 2019. In addition, only 410 articles were published in 2020, where the pandemic of COVID-19 and indexing delay may take the most responsibility (Figure 1). As for the article type, 10,443 publications were basic studies, which was accounted for 70.46% of the total publications. As a result, 1917 publications (12.93%) were reviews and meta analyzes, and other article types including clinical studies (1117 publications, 7.54%), comments, letters, or editorials (701 publications, 4.73%), abstracts (590 publications, 3.98%), and case reports (54 publications, 0.36%), which accounted for a relatively small proportion of the publications. The proportion of basic study and comment, letter, or editorial increased continuously over the past three decades, the proportion of abstract and case reports declined gradually, while the proportion of clinical studies and reviews and meta analyzes was basically unchanged (Figure 2).

**Figure 3:**
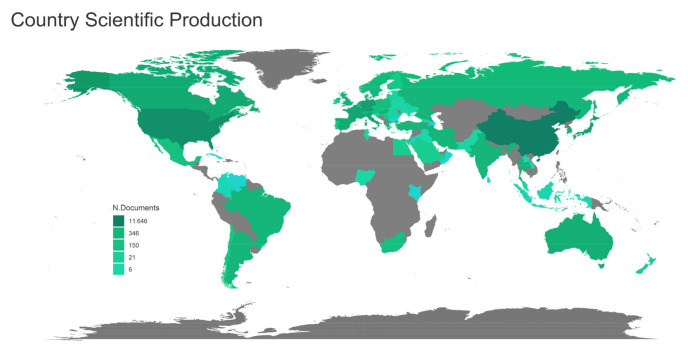
Articles published per country.

As shown in Figure 3, China, USA, Germany, Japan, and France were the top five countries with the greatest number of publications. Fourth Military Medical University, Chiang Mai University, Renmin Hospital of Wuhan University, Huazhong University of Science and Technology, and Central South University were the top five affiliations with the greatest number of publications. In addition, Cardiovascular Research, American Journal of Physiology-Heart and Circulatory Physiology, Circulation, Journal of Molecular and Cellular Cardiology, and Journal of Cardiovascular Pharmacology were the top five journals with the greatest number of publications. Details of the top 10 affiliations and journals with the most publications were listed in Table 1 and Table 2, respectively.

### 3.2. MeSH Analyses

MeSH terms with occurrence more than 25 times were included in MeSH analyses, and 1028 MeSH terms with a cumulative occurrence of 154,103 times were included in MeSH analyses.

**Figure 4:**
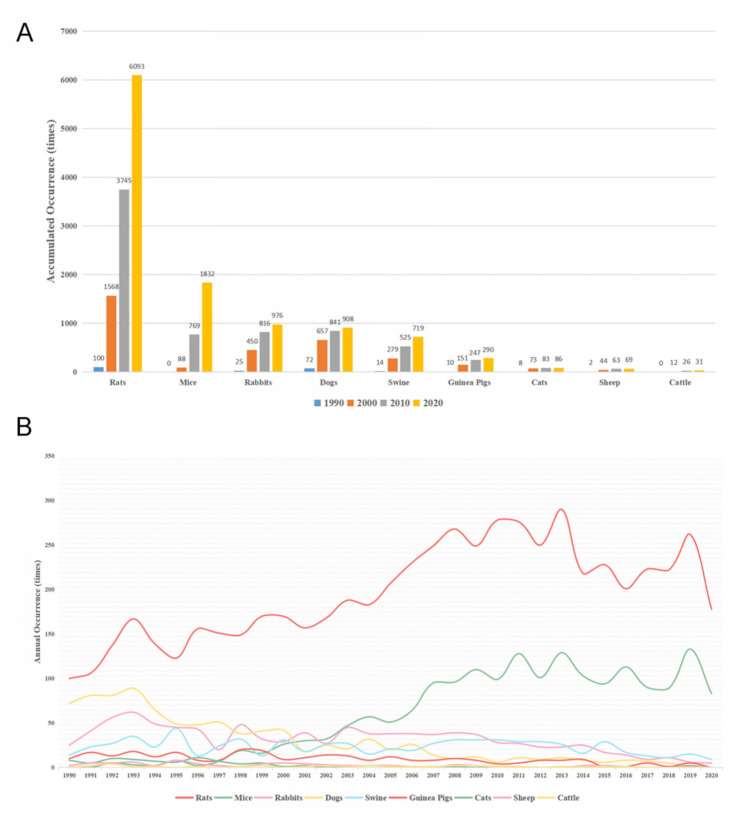
MeSH terms of animal types. (**A**) accumulated occurrence of MeSH terms every ten years; (**B**) annual occurrence of MeSH terms.

Animal was the major term of the research subject (12,601 times, 8.18%), followed by human (4521 times, 2.93%) and cell (848 times, 0.55%). Among the experimental animals, rats were most widely used in myocardial reperfusion injury, followed by mice, rabbits, dogs, and swine. The accumulated and annual occurrence of MeSH terms regarding animal types was shown in Figure 4 and the change rate of annual occurrence was presented in Supplementary Materials (Figure S1). These results showed that rats, mice, and rabbits were the most studied animals and the annual occurrence of these terms was basically unchanged except for 2020, where the reduction of total publication number may be the reason.

**Figure 5:**
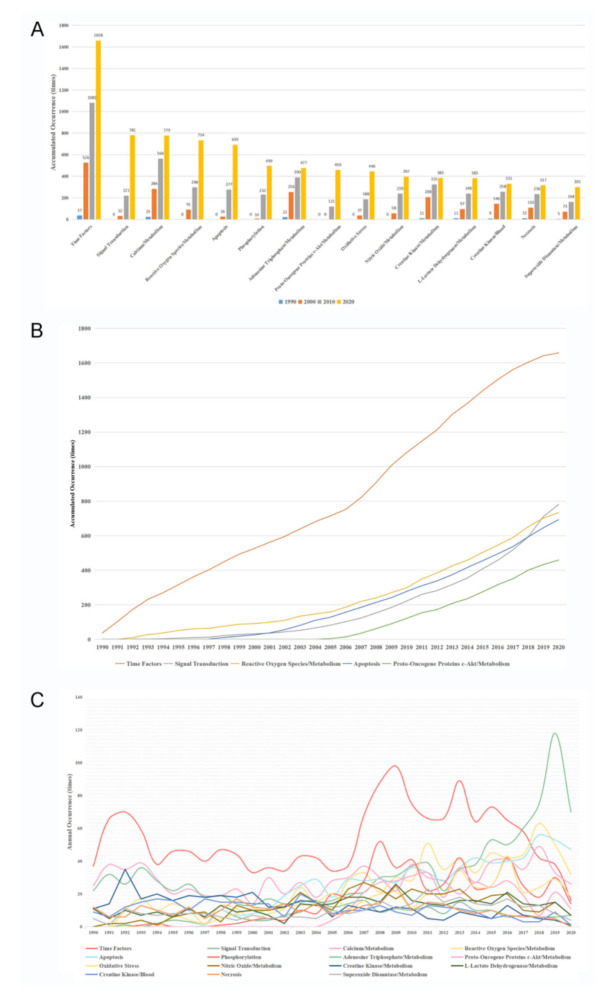
MeSH terms in relation to the pathogenesis of myocardial reperfusion injury. (**A**) accumulated occurrences of the top 15 MeSH terms every ten years; (**B**) accumulated occurrences of the top 5 MeSH terms with the greatest increasing trend; (**C**) annual occurrences of the top 15 MeSH terms.

Figure 5A showed the top 15 MeSH terms, which were closely associated with the pathogenesis of myocardial reperfusion injury. Time factor was the leading term (1658 times, 1.08%), followed by signal transduction (781 times, 0.51%), calcium/metabolism (779 times, 0.51%), reactive oxygen species/metabolism (734 times, 0.48%), and apoptosis (693 times, 0.45%). Since some of these terms were not indexed until 2005, we further calculated the average occurrence times of these terms from 2005 to 2020. The results showed that time factor (63 times/year), signal transduction (47 times/year), reactive oxygen species/metabolism (38 times/year), apoptosis (38 times/year), and proto-oncogene proteins c-Akt/metabolism (30 times/year) were the fastest increasing terms (Figure 5B). In addition, the annual occurrences of the top 15 MeSH terms were calculated and the occurrence of all these terms decreased in 2020, which may be caused by the reduced publication number. The term time factor shows a decreasing trend over the past decade, while signal transduction (118 times), apoptosis (53 times), and reactive oxygen species/metabolism (50 times) have become the top 3 MeSH terms in 2019 (Figure 5C). The change rate of annual occurrence was presented in Supplementary Materials (Figure S2).

**Figure 6:**
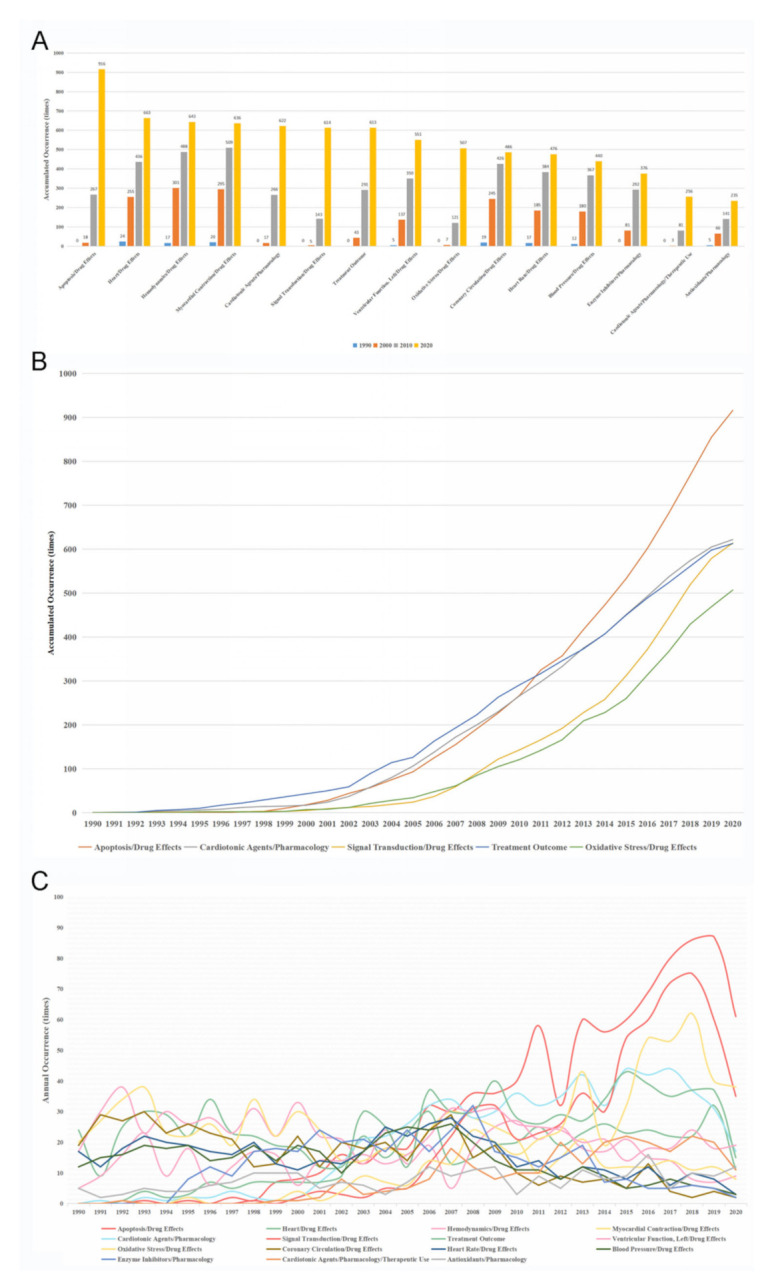
MeSH terms in relation to the treatment of myocardial reperfusion injury. (**A**) accumulated occurrences of the top 15 MeSH terms every ten years; (**B**) accumulated occurrences of the top 5 MeSH terms with the greatest increasing trend; (**C**) annual occurrences of the top 15 MeSH terms.

In order to further acknowledge the research situation concerning the treatment of myocardial reperfusion injury, the top 15 MeSH terms related to myocardial reperfusion injury treatment were identified (Figure 6A). Apoptosis/drug effects (916 times, 0.59%), heart/drug effects (66 times, 0.43%), hemodynamics/drug effects (643 times, 0.42%), myocardial contraction/drug effects (636 times, 0.41%), and cardiotonic agents/pharmacology (622 times, 0.40%) were the most concerning terms. Since some of these terms were not indexed until 1997, we further calculated the average occurrence times of these terms from 1997 to 2020. The results showed that apoptosis/drug effects (40 times/year), cardiotonic agents/pharmacology (27 times/year), signal transduction/drug effects (27 times/year), treatment outcome (26 times/year), and oxidative stress/drug effects (22 times/year) were the fastest increasing terms (Figure 6B). In addition, annual occurrences of the top 15 MeSH terms were calculated and the occurrences of most of these terms decreased in 2020, which may be caused by the reduced publication number. Apoptosis (87 times), signal transduction/drug effects (60 times), and oxidative stress/drug effects (40 times) were the top 3 MeSH terms in 2019. The change rate of annual occurrence was also presented in Supplementary Materials (Figure S3). In general, these results revealed the trend of research hot topics.

### 3.3. LDA Analyses

To obtain more detailed research topics, LDA analyses were performed based on the abstract of the publications, and a network was constructed to illustrate the relationship between these topics.

**Figure 7:**
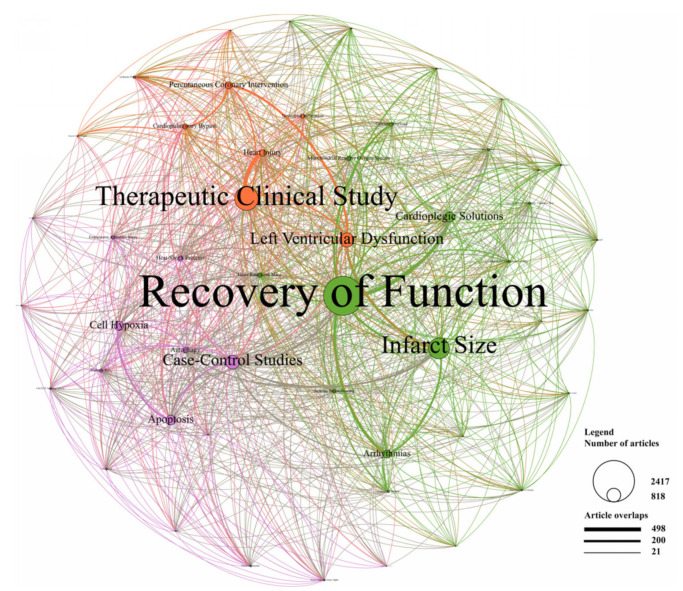
Topic cluster network by Latent Dirichlet allocation. The green region represents the cluster of prognosis, the purple region represents the cluster of mechanism, and the red region represents the cluster of treatment. The size of the circle represents the number of publications in each topic, and the degree of line thickness represents the weight of connections between topics.

As shown in Figure 7, 50 research topics were identified and classified into three clusters marked in different colors. The green region represented the cluster of prognosis, while the purple region and red region represented the cluster of mechanism and the cluster of treatment, respectively. The size of the circle represented the number of publications in each topic, while the degree of line thickness represented the weight of connection between topics.

Recovery of function, infarct size, cardioplegic solutions, arrhythmia, and gene knockout mice were the main topics in the cluster of prognosis. These topics showed a relatively strong correlation with each other, especially between cardioplegic solution and recovery of function. In the cluster of mechanism, case-control studies, cell hypoxia, apoptosis, autophagy, and heat-shock proteins were the top five topics. A relatively strong association was observed between cell hypoxia and apoptosis. Therapeutic clinical study was the predominant topic of the treatment cluster, followed by left ventricular dysfunction, percutaneous coronary intervention, heart injury, and cardiopulmonary bypass. A compact connection was detected between therapeutic clinical study and heart injury.

Viewing the network as a whole, recovery of function, therapeutic clinical study, infarct size, left ventricular dysfunction, and case-control studies were the most focused research topics. Topics in the cluster of prognosis accounted for about 50% of the total network, which indicated the prognosis was the most concerned field for scientists. There were also complex correlations between topics of different clusters, especially between left ventricular dysfunction and infarct size. In all, LDA analyses revealed the significance and connections of research topics.

## 4. Discussion

Myocardial ischemia is the major cause of death worldwide, and reperfusion is the standard intervention for myocardial ischemia. However, reperfusion may cause additional damage, known as myocardial reperfusion injury. Myocardial reperfusion injury was firstly identified in 1960 when Jennings and his team found that reperfusion could cause myocardial injury and increase infarct size in a dog model [20]. The prevention and treatment of myocardial reperfusion injury have become challenges for physicians ever since. This study, for the first time, comprehensively analyzed 14,822 publications in relation to myocardial reperfusion injury from 1990 to 2020 by machine learning. The number of studies in myocardial reperfusion injury fluctuated around 579 since 2006 while declined to 410 in 2020, which was caused by the pandemic of COVID-19 as well as index delay. Over 70% of the publications were basic studies, while clinical studies only accounted for a small proportion, indicating that more translational and clinical studies are encouraged in the future. In our study, we found that China and USA published the largest number of publications, with five and two of the top 10 affiliations with most publications, respectively. These countries and affiliations may continue to play significant roles in future publications for myocardial reperfusion injury. Cardiovascular Research was the journal with most publications, followed by American Journal of Physiology-Heart and Circulatory Physiology, Circulation, Journal of Molecular and Cellular Cardiology, and Journal of Cardiovascular Pharmacology. Additional studies about myocardial reperfusion injury are recommended to submit in these journals. In addition, four of the top 10 journals with most publications had an impact factor over 10, suggesting that researches on myocardial reperfusion injury were interesting and valuable.

MeSH analyses were applied to identify the trend of research hot topics [21]. Time factor was the leading term of the pathogenesis of myocardial reperfusion injury. It also remarkably increased over the past three decades. As we known, earlier reperfusion through advanced PCI technology and administration of more efficacious anti-platelet and anti-thrombotic agents are the most effective methods to reduce myocardial reperfusion injury [22]. In addition, myocardial reperfusion injury is time-dependent progress, started with increased oxidative stress, inflammation, calcium overload, and rapidly developed to apoptosis and cell death [23]. Interventions of these factors in different periods of the myocardial reperfusion injury may influence prognosis, which needs to be further studied. In addition, it seems that the researches of time factor have hit the bottleneck, resulting in the decrease of annual occurrence since 2015. Moreover, the research focus seems to be moved to signal transduction, apoptosis, and reactive oxygen species or oxidative stress in both pathogenesis and treatment researches, indicating more and more efforts have been made to transfer mechanism studies into clinical practice.

Since the MeSH term can only reflect the category of the publications rather than their specific contents [24], LDA analyses were used to obtain more detailed research topics from the abstract of the publications. Studies related to myocardial reperfusion injury were classified into three clusters: prognosis, mechanism, and treatment. The relationships among the clusters and the topics were demonstrated.

The prognosis of myocardial reperfusion injury, usually evaluated by the recovery of function, attracted the most attention in the past three decades. Infarct size is the major determinant of prognosis [25]. Lethal reperfusion injury caused by cardiomyocyte death at the end of the ischemic event was suggested to be responsible for up to 50% of the final infarct size [26]. Thus, it represents a significant target for improving the prognosis of patients with AMI treated with PCI. However, lethal reperfusion injury remains irreversible and lack of effective therapeutic strategies, which demands to be investigated urgently. Reperfusion-induced arrhythmia is widely recognized as a reversible injury that can self-terminate or can be easily treated pharmacologically, according to the type of arrhythmia. However, recent studies found that reperfusion-induced arrhythmia may also result in cardiomyocyte death and contribute to the final infarct size [27,28,29]. Therefore, van der Weg K, et al. recommended that reperfusion-induced arrhythmia should be recognized as a marker of lethal reperfusion injury rather than an independent event [30]. This point of view updates our understanding of reperfusion injury, suggesting that the previous classification may be inaccurate to reflect the complex process of myocardial reperfusion injury. Cardioplegic solutions were continuously applied to reduce myocardial reperfusion injury and improve functional recovery in cardiac surgery, despite adverse effects on endothelial cells [31]. Recent studies suggest that the application of compounds improving nitric oxide (NO) bioavailability may be an effective strategy to protect both endothelial cells and cardiomyocytes [32], which demands more preclinical studies and toxicity assessment to apply these compounds into the clinic.

The research hotspots of mechanism in myocardial reperfusion injury mainly include oxidative stress, inflammation, calcium overload, and energy metabolism disorders. These factors are interrelated with each other and finally result in cardiomyocyte death. Unfortunately, therapeutic interventions targeting these factors had little effect on the prevention and treatment of myocardial reperfusion injury [22]. Therefore, the identification of new mechanisms and potential therapeutic targets are paramount. Several therapeutic strategies have been proposed and proved to be effective in experimental studies, such as ischemic pre-, per-, and post-conditioning [5], therapeutic hyper-oxemia [6], and traditional Chinese herbal medicine [4]. However, there is poor evidence in regard to translation of these strategies into the clinic, which may be caused by an incomplete understanding of their underlying mechanisms. Additional investigations are required on the mechanism and treatment of myocardial reperfusion injury.

This study, indeed, bore several limitations. Firstly, PubMed was searched for publications related to myocardial reperfusion injury because it contains the highest quality of publications and rules out irrelevant, non-peer-reviewed publications. However, this database does not provide citation information and analyses of the highly-cited publications are not available. Combined analyses of other databases may provide more information. Secondly, MeSH terms were used to search the related publications, thus those that have not been indexed by MeSH were not included in this study. Lastly, further bibliometric analyses can be applied to other types of topics such as neurodegenerative diseases, cancer, and metabolic syndrome.

## 5. Conclusions

The number of publications on myocardial reperfusion injury increases during the past three decades, which mainly focused on prognosis, mechanism, and treatment. Complex correlations are observed between topics of different clusters. Prognosis is the most concerned field, while studies on mechanism and treatment are relatively less to be investigated.

## Figures and Tables

**Table 1:** Top 10 affiliations with most publications related to myocardial reperfusion injury.

Affiliation	Country	Documents
Fourth Military Medical University	China	814
Chiang Mai University	Thailand	376
Renmin Hospital of Wuhan University	China	352
Huazhong University of Science and Technology	China	349
Central South University	China	266
University of California	USA	251
University College London	UK	244
Capital Medical University	China	226
Medical College of Wisconsin	USA	223
University of Alberta	Canada	202

**Table 2:** Top 10 journals with most publications related to myocardial reperfusion injury.

Journal Title	Country	Documents	IF 2020
Cardiovascular Research	UK	661	10.787
American Journal of Physiology-Heart and Circulatory Physiology	USA	587	4.733
Circulation	USA	456	26.690
Journal of Molecular and Cellular Cardiology	UK	417	5.000
Journal of Cardiovascular Pharmacology	USA	363	3.105
Annals of Thoracic Surgery	USA	303	4.330
Basic Research in Cardiology	Germany	299	17.165
Journal of Thoracic and Cardiovascular Surgery	USA	285	5.209
European Journal of Pharmacology	Netherlands	238	4.432
Circulation Research	USA	203	17.367

## Data Availability

All data generated or analyzed during this study are included in this published article.

## Supplementary Materials

The following are available online at https://www.mdpi.com/article/10.3390/ijerph18158231/s1, Table S1: Relatived codes; Figure S1: Change rate of the MeSH terms related to animal types; Figure S2: Change rate of the top 15 MeSH terms related to the pathogenesis of myocardial reperfusion injury.; Figure S3: Change rate of the top 15 MeSH terms related to the treatment of myocardial reperfusion injury.

## References

[1] Lillo-Moya J., Rojas-Solé C., Muñoz-Salamanca D., Panieri E., Saso L., Rodrigo R. (2021). Targeting Ferroptosis against Ischemia/Reperfusion Cardiac Injury. Antioxidants.

[2] Schanze N., Bode C., Duerschmied D. (2019). Platelet Contributions to Myocardial Ischemia/Reperfusion Injury. Front. Immunol..

[3] Yellon D.M., Hausenloy D.J. (2007). Myocardial reperfusion injury—Reply. N. Engl. J. Med..

[4] Wang R., Wang M., Zhou J., Wu D., Ye J., Sun G., Sun X. (2020). Saponins in Chinese Herbal Medicine Exerts Protection in Myocardial Ischemia-Reperfusion Injury: Possible Mechanism and Target Analysis. Front. Pharmacol..

[5] Davidson S.M., Ferdinandy P., Andreadou I., Bøtker H.E., Heusch G., Ibáñez B., Ovize M., Schulz R., Yellon D.M., Hausenloy D.J. (2019). Multitarget Strategies to Reduce Myocardial Ischemia/Reperfusion Injury: JACC Review Topic of the Week. J. Am. Coll. Cardiol..

[6] O’Neill W.W., Martin J.L., Dixon S.R., Bartorelli A.L., Trabattoni D., Oemrawsingh P.V., Atsma D.E., Chang M., Marquardt W., Oh J.K. (2007). Acute Myocardial Infarction with Hyperoxemic Therapy (AMIHOT): A prospective, randomized trial of intracoronary hyperoxemic reperfusion after percutaneous coronary intervention. J. Am. Coll. Cardiol..

[7] Zhou H., Tan W., Qiu Z., Song Y., Gao S.A. (2018). Bibliometric analysis in gene research of myocardial infarction from 2001 to 2015. PeerJ.

[8] Iftikhar P.M., Uddin M.F., Ali F., Arastu A.H., Khan J., Munawar M., Suleman J. (2020). The Top Most-Cited and Influential Published Articles in Atrial Fibrillation from 1900 to 2019. Am. J. Cardiol..

[9] Devos P., Menard J. (2019). Bibliometric analysis of research relating to hypertension reported over the period 1997–2016. J. Hypertens..

[10] Chang C., Gau M., Tang K., Hwang G. (2021). Directions of the 100 most cited nursing student education research: A bibliometric and co-citation network analysis. Nurs. Educ. Today.

[11] Brandt J.S., Hadaya O., Schuster M., Rosen T., Sauer M.V., Ananth C.V. (2019). A Bibliometric Analysis of Top-Cited Journal Articles in Obstetrics and Gynecology. JAMA Network. Open.

[12] Ahmad T., Nasir S., Musa T.H., AlRyalat S., Khan M., Hui J. (2021). Epidemiology, diagnosis, vaccines, and bibliometric analysis of the 100 top-cited studies on Hepatitis E virus. Hum. Vaccin. Immunother..

[13] Chen X., Xie H., Wang F.L., Liu Z., Xu J., Hao T. (2018). A bibliometric analysis of natural language processing in medical research. BMC Med. Inform. Decis. Mak..

[14] Wang K., Feng C., Li M., Pei Q., Li Y., Zhu H., Song X., Pei H., Tan F. (2020). A bibliometric analysis of 23,492 publications on rectal cancer by machine learning: Basic medical research is needed. Therap. Adv. Gastroenterol..

[15] Stout N.L., Alfano C.M., Belter C.W., Nitkin R., Cernich A., Lohmann S.K., Chan L. (2018). A Bibliometric Analysis of the Landscape of Cancer Rehabilitation Research (1992–2016). J. Natl. Cancer Inst..

[16] Aria M., Cuccurullo C. (2017). Bibliometrix: An R-tool for comprehensive science mapping analysis. J Informetr..

[17] Feng C., Wu Y., Gao L., Guo X., Wang Z., Xing B. (2019). Publication Landscape Analysis on Gliomas: How Much Has Been Done in the Past 25 Years?. Front. Oncol..

[18] Dozmorov M.G. (2018). GitHub Statistics as a Measure of the Impact of Open-Source Bioinformatics Software. Front. Bioeng. Biotech..

[19] Jacomy M., Venturini T., Heymann S., Bastian M. (2014). ForceAtlas2, a continuous graph layout algorithm for handy network visualization designed for the Gephi software. PLoS ONE.

[20] Jennings R.B., Sommers H.M., Smyth G.A., Flack H.A., Linn H. (1960). Myocardial necrosis induced by temporary occlusion of a coronary artery in the dog. Arch. Pathol..

[21] Zhao F., Shi B., Liu R., Zhou W., Shi D., Zhang J. (2018). Theme trends and knowledge structure on choroidal neovascularization: A quantitative and co-word analysis. BMC Ophthalmol..

[22] Hausenloy D.J., Yellon D.M. (2013). Myocardial ischemia-reperfusion injury: A neglected therapeutic target. J. Clin. Invest..

[23] Neri M., Riezzo I., Pascale N., Pomara C., Turillazzi E. (2017). Ischemia/Reperfusion Injury following Acute Myocardial Infarction: A Critical Issue for Clinicians and Forensic Pathologists. Mediat. Inflamm..

[24] Liu J., Wang L., Wang Z., Liu J. (2019). Roles of Telomere Biology in Cell Senescence, Replicative and Chronological Ageing. Cells.

[25] Heusch G. (2020). Myocardial ischaemia–reperfusion injury and cardioprotection in perspective. Nat. Rev. Cardiol..

[26] Griffiths K., Lee J.J., Frenneaux M.P., Feelisch M., Madhani M. (2021). Nitrite and myocardial ischaemia reperfusion injury. Where are we now?. Pharmacol. Ther..

[27] van der Weg K., Majidi M., Haeck J.D., Tijssen J.G., Green C.L., Koch K.T., Kuijt W.J., Krucoff M.W., Gorgels A.P., de Winter R.J. (2016). Ventricular arrhythmia burst is an independent indicator of larger infarct size even in optimal reperfusion in STEMI. J. Electrocardiol..

[28] van der Weg K., Kuijt W.J., Bekkers S.C., Tijssen J.G., Green C.L., Lemmert M.E., Krucoff M.W., Gorgels A.P. (2018). Reperfusion ventricular arrhythmia bursts identify larger infarct size in spite of optimal epicardial and microvascular reperfusion using cardiac magnetic resonance imaging. Eur. Heart J. Acute Cardiovasc. Care.

[29] Majidi M., Kosinski A.S., Al-Khatib S.M., Smolders L., Cristea E., Lansky A.J., Stone G.W., Mehran R., Gibbons R.J., Crijns H.J. (2015). Implications of ventricular arrhythmia “bursts” with normal epicardial flow, myocardial blush, and ST-segment recovery in anterior ST-elevation myocardial infarction reperfusion: A biosignature of direct myocellular injury “downstream of downstream”. Eur. Heart J. Acute Cardiovasc. Care.

[30] van der Weg K., Prinzen F.W., Gorgels A.P.M. (2019). Editor’s Choice- Reperfusion cardiac arrhythmias and their relation to reperfusion-induced cell death. Eur. Heart J. Acute Cardiovasc. Care.

[31] Yang Q., He G. (2005). Effect of Cardioplegic and Organ Preservation Solutions and Their Components on Coronary Endothelium-Derived Relaxing Factors. Ann. Thorac. Surg..

[32] Pisarenko O., Studneva I. (2021). Modulating the Bioactivity of Nitric Oxide as a Therapeutic Strategy in Cardiac Surgery. J. Surg. Res..

