# Supplementary material for: A Bibliometric Analysis of 14,822 Researches on Myocardial Reperfusion Injury by Machine Learning
Source: Int J Environ Res Public Health. 2021 Aug 3;18(15):8231. doi: 10.3390/ijerph18158231 (PMC8345983; doi:10.3390/ijerph18158231)
Supplement: Supplementary file 1 [file ijerph-18-08231-s001.zip › ijerph-1311814-supplementary.pdf]

International Journal of
Environmental Research and Public Health

MDPI

Supplementary Material. Table S1-Relatived codes

```
from nltk.tokenize import RegexpTokenizer
from stop_words import get_stop_words
from nltk.stem.porter import PorterStemmer
from sklearn.utils import shuffle
from gensim import corpora, models
import pandas as pd
import logging
import pickle
import numpy as np
logging.basicConfig(level = logging.INFO,format = '%(asctime)s - %(name)s - %(levelname)s - %(message)s')
logger = logging.getLogger(__name__)


from gensim.models.ldamulticore import LdaMulticore

def dumppick(filename):
    corpus = []
    tokens = []
    df = pd.read_csv(filename,sep=',',encoding="utf8")
    df = df[df["abstract"].isna()!=True]
    #df = df[df.year<2019]

    for line in df["abstract"]:
        corpus.append(line.strip())
    del df

    stop_words = get_stop_words('english')
    en_stop = [ str(i).strip() for i in stop_words ]


    p_stemmer = PorterStemmer()


    print("wen ben yu chu li")
    tokenizer = RegexpTokenizer(r'[A-Za-z]+')
    for i,text in enumerate(corpus):
        if i%1000==0:
            print(i)
        raw = text.lower()
        token = tokenizer.tokenize(raw)
        stop_remove_token = [word for word in token if (word not in en_stop and len(word)>1)]
        stem_token = [p_stemmer.stem(word) for word in stop_remove_token]
        tokens.append(stop_remove_token)
    # print tokens

    print("start" )
    dictionary = corpora.Dictionary(tokens)
    # print dictionary.token2id
    # print type(dictionary)
    print("")
    texts = [dictionary.doc2bow(text) for text in tokens]
    print("accomplished")
```

```
    # lda_model = models.ldamodel.LdaModel(texts, num_topics=3, id2word=dictionary,    passes=500)
    # print lda_model.print_topics(num_topics=3,num_words=4)
    # corpus_lda = lda_model[texts]
    # for doc in corpus_lda:
    #       print doc


    print("开始  tfidf")
    texts_tf_idf = models.TfidfModel(texts)[texts]
    # # for text in texts_tf_idf:
    # #       print text
    # lda_tf_idf = models.LdaModel(texts_tf_idf, num_topics=3, id2word=dictionary, update_every=0, passes=200)
    # print lda_tf_idf.print_topics(num_topics=3,num_words=4)
    # # doc_topic = [a for a in lda_tf_idf[texts_tf_idf]]
    # # for topic_id in range(3):
    # #       print "topic:{}".format(topic_id+1)
    # #       print lda_tf_idf.show_topic(topic_id)
    # corpus_lda_tfidf = lda_tf_idf[texts_tf_idf]
    # for doc in corpus_lda_tfidf:
    #       print doc
    pickle.dump(texts, open("text_dtm.pickle","wb"))
    pickle.dump(texts_tf_idf, open("texts_tf_idf_dtm.pickle","wb"))
    pickle.dump(dictionary, open("dictionary.pickle","wb"))




def loadpcik():
    texts = pickle.load(open("text_dtm.pickle","rb"))
    texts_tf_idf = pickle.load(open("texts_tf_idf_dtm.pickle","rb"))
    dictionary = pickle.load(open("dictionary.pickle","rb"))
    return texts, texts_tf_idf,dictionary

#dumppick()
def createlda(num_topics,filename):
    dumppick(filename)
    num_topics = 50
    texts, texts_tf_idf, dictionary = loadpcik()

    """
    print("**************LSI*************")
    lsi = models.lsimodel.LsiModel(corpus=texts, id2word=dictionary, num_topics=20)
    texts_lsi = lsi[texts_tf_idf]
    print(lsi.print_topics(num_topics=20, num_words=10))
    """

    print("**************LDA*************")
    #ppl = []
    #for i in range(1,50,1):
        #texts = shuffle(texts)
```

```
        #texts_train = texts[:int(24012*(0.9))]
        #texts_vad = texts[int(24012*(0.9)):]
    lda = LdaMulticore(corpus=texts,iterations=1000, id2word=dictionary, num_topics=num_top-
ics,passes=200,per_word_topics=True)
    #texts_lda = lda[texts_tf_idf]
    out = open("ldamd{}tpc-tpc".format(num_topics),"w",encoding ="utf8")
    print(lda.print_topics(num_topics=num_topics, num_words=10),file =out)
    lda.save("ldamd{}tpc+{}".format(num_topics,filename[9:18]))
        #ppl.append(np.exp2(-lda.log_perplexity(texts_vad))/i)
    return lda,texts, texts_tf_idf, dictionary


def lodaldaml():
    lda = models.LdaModel.load("ldamd50tpc-tpc")

def saveldatpcw(lda):
    tpcn = 50
    tpcw = pd.DataFrame(columns=[i for i in range(1,11)])
    for i in range(tpcn):
        tpcw.loc[i] = [ w for w,p in lda.show_topic(i)]
    tpcw.to_csv("newdatatpcw.csv")


def get_cite_n_dmt(dictionary,citenum=0,):
    citenum=0
    corpus = []
    tokens = []
    df = pd.read_csv("pubmed2csv.csv",sep=',',encoding="utf8")
    df = df[df["施引文献"]==citenum]
    df = df[df["abstract"].isna()!=True]

    for line in df["abstract"]:
        corpus.append(line.strip())
    del df

    stop_words = get_stop_words('english')
    en_stop = [ str(i).strip() for i in stop_words ]

    p_stemmer = PorterStemmer()

       print("wen ben yu chu li")
    tokenizer = RegexpTokenizer(r'[A-Za-z]+')
    for i,text in enumerate(corpus):
        if i%1000==0:
            print(i)
        raw = text.lower()
        token = tokenizer.tokenize(raw)
        stop_remove_token = [word for word in token if (word not in en_stop and len(word)>1)]
        stem_token = [p_stemmer.stem(word) for word in stop_remove_token]
        tokens.append(stop_remove_token)
    texts_cite_n = [dictionary.doc2bow(text) for text in tokens]
    return texts_cite_n

def get0cited2tpc(lda,texts):
```

```
    tpc1 = []
    tpc2 = []
    for i in texts_cite_0:
        tpc = lda.get_document_topics(i)
        tpc = sorted(tpc,key=lambda x:-x[1])
        tpc1.append(tpc[0][0])
        if len(tpc)>1:
            tpc2.append(tpc[1][0])
        else:
            tpc2.append(lda.num_topics+1)
    df = pd.read_csv("withoutNAabstract",sep=',',encoding="utf8")
    df = df[df["reference"]==0]
    df = df[df["abstract"].isna()!=True]
    df["tpc1"] = tpc1
    df["tpc2"] = tpc2
    df.to_csv("0cite_tpc.csv")
    return tpc1,tpc2

def getallcited2tpc(lda,texts,filename):
    tpc1 = []
    tpc2 = []
    for i in texts:
        tpc = lda.get_document_topics(i)
        tpc = sorted(tpc,key=lambda x:-x[1])
        tpc1.append(tpc[0][0])
        if len(tpc)>1:
            tpc2.append(tpc[1][0])
        else:
            tpc2.append(lda.num_topics+1)
    df = pd.read_csv(filename,sep=',',encoding="utf8")
    df = df[df["abstract"].isna()!=True]
    #df = df[df.year<2019]
    df["tpc1"] = tpc1
    df["tpc2"] = tpc2
    df.to_csv(filename)
    return tpc1,tpc2

def grap(tpc1,tpc2,tpcn,filename):
    from collections import    Counter
    CC = Counter(tpc1)
    import networkx as nx
    G = nx.Graph()
    for i in range(tpcn):
        G.add_node(i,num=CC[i])
    #edgscount = Counter([(i,j) for i,j in zip(tpc1,tpc2)])
    #for edgs,count in edgscount.items()
    edgeslist = [(i,j)    for i,j in zip(tpc1,tpc2) if j<tpcn]
    G.add_edges_from(edgeslist,Weight=0)
    for i,j in zip(tpc1,tpc2):
        if j >= tpcn:
            continue
        G.edges[i,j]["Weight"]+=1
    nx.write_graphml(G,filename.replace(".csv",".graphml"),encoding="utf8")
    return G
```

```
def Grap_Add_tpcname():
    import pandas as pd
    c = pd.read_excel("newdatatpcw.xlsx")
    pic = {tpcid:name for tpcid,name in  zip( range(50),c["meshtermnaming"])}
    import networkx as nx
    g = nx.read_graphml("withoutNAabstract.graphml")
    for i in range(50):
        g.node[str(i)]["name"] = pic[i]
    nx.write_graphml(g,"withoutNAabstract_addname.graphml",encoding="utf8")

def main():
    filename = "withoutNAabstract.csv"

    lda,texts, texts_tf_idf, dictionary  = createlda(50,filename)

    saveldatpcw(lda)

    tpc1,tpc2 = getallcited2tpc(lda,texts,filename)

    grap(tpc1,tpc2,50,filename)

    Grap_Add_tpcname()

if __name__ == "__main__":
    main()
```

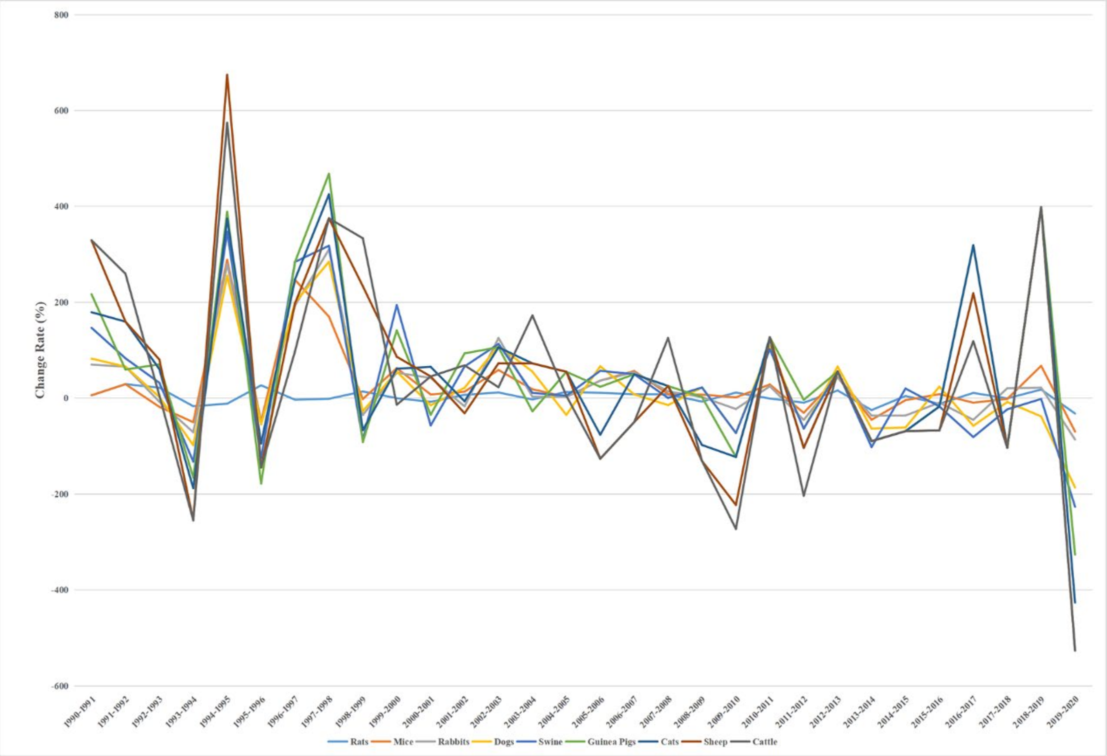

Figure S1. Change rate of the MeSH terms related to animal types.

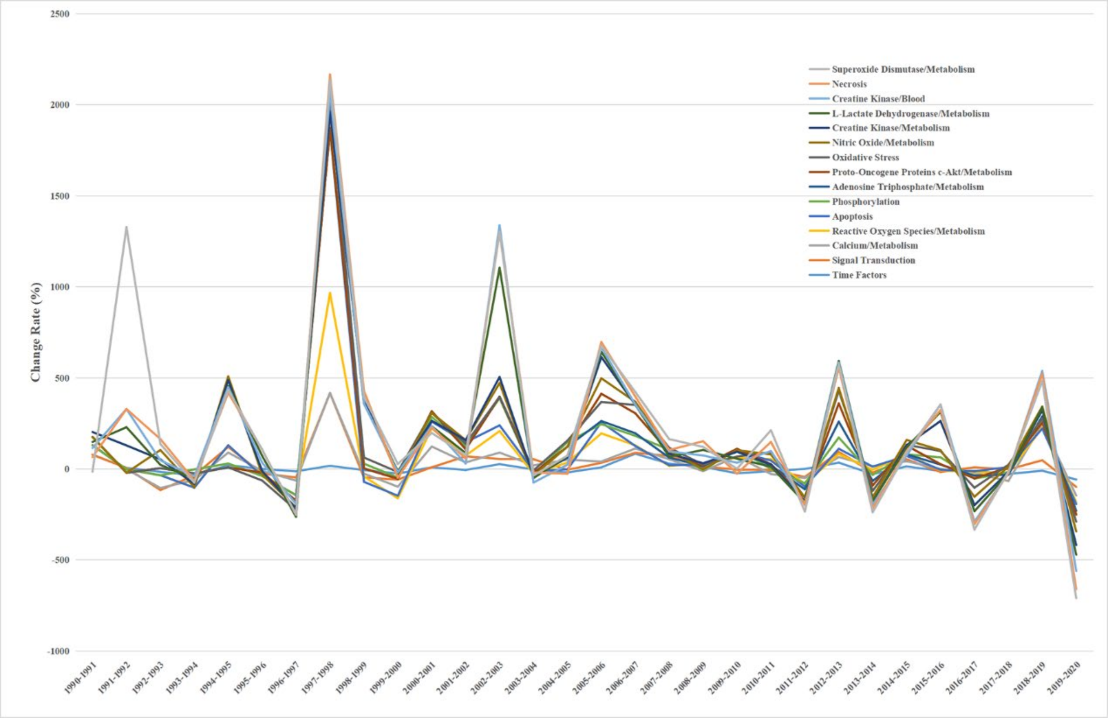

Figure S2. Change rate of the top 15 MeSH terms related to the pathogenesis of myocardial reperfusion injury.

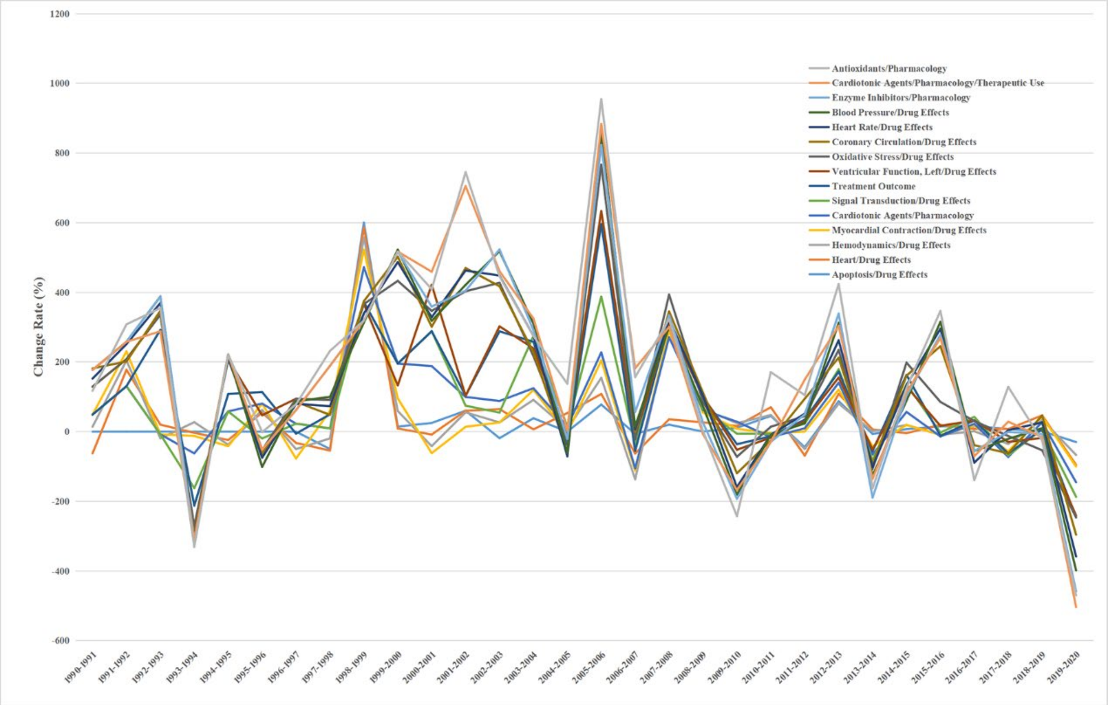

Figure S3. Change rate of the top 15 MeSH terms related to the treatment of myocardial reperfusion injury.
